# AliClu - Temporal sequence alignment for clustering longitudinal clinical data

**DOI:** 10.1186/s12911-019-1013-7

**Published:** 2019-12-30

**Authors:** Kishan Rama, Helena Canhão, Alexandra M. Carvalho, Susana Vinga

**Affiliations:** 1 Instituto de Telecomunicações, Instituto Superior Técnico, Universidade de Lisboa, Avenida Rovisco Pais, 1 - Torre Norte Piso 10., Lisboa, 1049-001 Portugal; 2 CEDOC, EpiDoC Unit, NOVA Medical School, National School of Public Health, Universidade NOVA de Lisboa, Rua do Instituto Bacteriológico, n∘ 5 Lab 2.9., Lisboa, 1150-082 Portugal; 3 INESC-ID, Instituto Superior Técnico, Universidade de Lisboa, Rua Alves Redol 9, Lisboa, 1000-029 Portugal

**Keywords:** Temporal sequence alignment, Clustering, Bootstrap, clustering indices

## Abstract

**Background:**

Patient stratification is a critical task in clinical decision making since it can allow physicians to choose treatments in a personalized way. Given the increasing availability of electronic medical records (EMRs) with longitudinal data, one crucial problem is how to efficiently cluster the patients based on the temporal information from medical appointments. In this work, we propose applying the Temporal Needleman-Wunsch (TNW) algorithm to align discrete sequences with the transition time information between symbols. These symbols may correspond to a patient’s current therapy, their overall health status, or any other discrete state. The transition time information represents the duration of each of those states. The obtained TNW pairwise scores are then used to perform hierarchical clustering. To find the best number of clusters and assess their stability, a resampling technique is applied.

**Results:**

We propose the AliClu, a novel tool for clustering temporal clinical data based on the TNW algorithm coupled with clustering validity assessments through bootstrapping. The AliClu was applied for the analysis of the rheumatoid arthritis EMRs obtained from the Portuguese database of rheumatologic patient visits (Reuma.pt). In particular, the AliClu was used for the analysis of therapy switches, which were coded as letters corresponding to biologic drugs and included their durations before each change occurred. The obtained optimized clusters allow one to stratify the patients based on their temporal therapy profiles and to support the identification of common features for those groups.

**Conclusions:**

The AliClu is a promising computational strategy to analyse longitudinal patient data by providing validated clusters and by unravelling the patterns that exist in clinical outcomes. Patient stratification is performed in an automatic or semi-automatic way, allowing one to tune the alignment, clustering, and validation parameters. The AliClu is freely available at https://github.com/sysbiomed/AliClu.

## Background

The increasing availability of clinical data and the increased investments in healthcare are driving research on building better clinical decision support systems for the effective personalization of treatments. In this context, machine learning and data mining techniques are becoming ubiquitous, helping to provide high-quality care systems and improve the long-term health of patients.

Patients’ health records are being stored in electronic medical records (EMRs) and consist of a variety of data, such as demographics, medical history, laboratory test results, medications, and allergies. These EMR systems are designed to store patients’ data across time, thereby providing large longitudinal cohorts. Exploring the disease heterogeneity and patterns in these datasets is a challenging task. Several issues contribute to this difficulty of this task: the exponential number of all possible combinations in patients’ trajectories, the variability in their temporal scales, and the complexity of their representations.

We address the problem of learning temporal patterns in EMR data by using a combined approach of (temporal) alignment and hierarchical clustering. More specifically, we use the Temporal Needleman-Wunsch (TNW) algorithm [1] to align discrete sequences with the time information between symbols and, subsequently, perform hierarchical clustering using the obtained pairwise scores. The TNW algorithm is an extension of the traditional Needleman-Wunsch (NW) [2] for global sequence alignment. The TNW takes into account the matches between symbols, as in the NW algorithm, and also adds a penalization term for the differences in the time values between two sequences. Other temporal alignment methods, such as dynamic time warping, are not adequate for dealing with these type of data, and they just provide general trends for matching continuous-time signals [3–6].

The TNW is particularly interesting when utilizing data representing given events or states (coded as symbols) and their corresponding durations. Treatment switching provides us with an excellent example of this type of temporal sequence data. Starting at instant 0 with Treatment A, its failure after *t*_*A*_ may lead to switching to Treatment B with a duration of *t*_*B*_, and then switching again to Treatment *F*, which is still ongoing (*t*_*F*_ represents that duration). In this case, we would have a patient profile given by the sequence

$$0.A,t_{A}.B,t_{B}.F,t_{F}.Z,$$

which includes symbols and numeric values and where *Z* is a special symbol representing that the last therapy has not yet failed. It is worth noting that the discrete states (*A*, *B* and *F* in this example) can also be obtained through the discretization of the continuous features. Additionally, the times representing the durations of the states are completely general with the only restriction being that they are measured at the same scale for all patients.

State-of-the-art alignment approaches usually involve multiple sequence alignment techniques that use the progressive alignment heuristic: they are fast, scalable and widely used. The most popular methods include Clustal Omega [7], MAFFT [8], and MUSCLE [9]. These methods were essentially developed for aligning DNA or protein sequences, which are time-invariant sequences composed by letters.

In this work, we focus specifically on using the temporal information present in clinical data for pairwise sequence alignment. In this regard, the literature includes mostly alignment algorithms for continuous time series data [4–6]. A very well known approach is Dynamic Time Warping (DTW) [3], which warps the time axis of the sequences to achieve alignment. It is also based on dynamic programming, such as the NW algorithm [2], but it does not incorporate a gap penalty. Pairwise alignment using Hidden Markov Models (HMMs) also constitutes an alternative [10]; however, it is not trivial to directly include temporal data.

Motivated by the need for a sequence alignment method that can assess the similarity between two sequences in the same way as the NW or HMM does while also accounting for the time that elapses between events, Syed and Das developed the TNW algorithm [1] that can be applied to healthcare data to find similar patients based on medical histories.

An alternative approach could be simply applying traditional sequence alignments such as the NW to sequences after some pre-processing step. This step would account for the temporal information between events by repeating an event several times to create the sequences to be aligned. For example, the temporal sequence "0.A,5.B" could be transformed to "AAAAAB", where the five As in the latter sequence represent the five units of time that elapsed from "A" to "B". Then, the NW algorithm can be applied. Several drawbacks exist in this approach; namely, the need to divide the time intervals between events in windows and the longer sequences that are created, thus increasing the computational time of the alignments. The TNW algorithm overcomes these issues and does not require any additional transformation of the original data. The absence of related works in the literature on this algorithm motivated us to test it on the Reuma.pt dataset [11].

The main goal of this work is to obtain clusters of patients by analysing longitudinal medical data specifically, clinical data. Clustering patients with similar treatment profiles would allow for identifying the common features of those groups and delineate strategies to improve treatment outcomes.

In the literature, several studies are found that try to achieve the same objective. In [12], Docampo et al. present a cluster analysis of clinical data to identify fibromyalgia subgroups. Their approach is a two-step clustering process. In the first step, the clinical variables are clustered by using partitioning around medoids. The number of clusters is found by using silhouette plots and Calinski’s index. In the second step, synthetic patient indices are calculated for each sample and dimension in order to find the patient subgroups.

In another work [13], Garg et al. proposed two techniques based on survival trees to cluster patients into clinically meaningful groups according to their expected lengths of stay. Their techniques are more applicable to survival analysis using survival data.

In [14], the authors investigated if subgroups of patients with non-specific lower back pain could be identified by applying hierarchical cluster analysis to a dataset that contained 6-month clinical courses of patients with measurements of bothersomeness. An initial step was required before using the clustering algorithm, which consisted of condensing the courses of each patient based on four parameters. These parameters were obtained by fitting a regression line in the courses and computing the slopes and intersections. After the parameters were defined for each patient, hierarchical clustering utilizing Ward’s method was applied. In order to determine the optimal number of clusters, they analysed the resulting dendrograms with Calinski’s criterion, which was also used in [12]. Regarding the results, four clusters were found with distinct clinical courses, which showed that it is possible to find clinical meaningful clusters based on the temporal evolution of the variable under study. Note that, in this work, the temporal information between measurements is not directly used, but we estimate the parameters of a line that is fit for the clinical courses.

In addition to the clustering approaches discussed before, a model-based clustering method was proposed for clustering individuals based on measurements taken over time [15]. The authors apply their method to data from pregnant women to identify hormone trajectories. One important aspect of this approach is that the method requires the specification of the number of clusters to be fit to the model. In their work, it was known that data were divided into two groups; hence, they knew the number of clusters to select.

However, this number was also confirmed by the Bayesian information criterion that they used to choose the number of clusters.

To the best of our knowledge, the AliClu is a novel approach for addressing this type of mixed, longitudinal data that takes into account both the sequence of states and their durations. The TNW algorithm allows one to align similar medical histories by considering the temporal information and also penalising missing events by inserting gaps. Furthermore, the AliClu provides clustering validation using bootstrapping, which allows one to tune the input parameters to find the best number of clusters and to identify the most homogeneous patient strata. The AliClu is fully implemented and freely available for further applications.

## Implementation

**Fig. 1:**
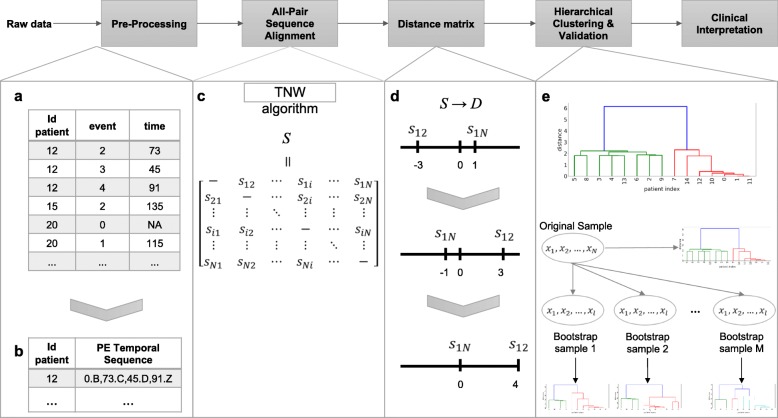
The proposed AliClu approach. First, raw data is pre-processed to obtain PE sequences. Then, pairwise sequence alignment is performed and a similarity matrix *S* is obtained. Next, *S* is converted into a distance matrix *D*. Agglomerative clustering is then performed with this distance matrix *D*. Validation of the clustering results is accomplished via a bootstrapping approach. In the end, retrieved clusters are analysed by the clinicians

The pipeline of the proposed method, which is named the AliClu, is illustrated in Fig. 1. In the first step, the complete raw data are pre-processed to obtain the temporal sequences. Then, in the second step, pairwise temporal sequence alignment is performed, and a similarity matrix is obtained. The third step consists of converting the similarity matrix into distances. Agglomerative clustering is then performed by using this distance matrix, and finally, the clustering results are validated via a bootstrapping approach. The obtained patient stratification can be graphically represented to ease the clinical interpretation. Each step of this pipeline is detailed as follows.

### Data pre-processing

This pre-processing step creates temporal sequences for each patient from EMRs. Patients’ records are typically available in *panel data* format, in which each patient is spread in different lines, one for each medical appointment, and the columns contain the features of interest measured over time. In this work, we consider that each patient experiences a sequence of events over time. Let A and B be the events of interest for a given patient with the time-distance *t* between them, and a *prefix-encoded* (PE) sequence for that patient is defined as 0.*A,t*.*B*.

In this pre-processing phase, the PE sequences are built for each patient, requiring information about the patient’s ID, the event under study, and the time between two consecutive events. These features must be taken from the panel data. In the data set, the time may be formatted as a date or just a number in any time unit (e.g., seconds, minutes, or days). Depending on the time format, two types of pre-processing steps are implemented. We refer the interested reader to the Additional file 1 for further details.

### Temporal sequence alignment

After building the prefix-encoded (PE) sequences, it is possible to align all patient pairs using the TNW algorithm [1]. The TNW guarantees convergence to the optimal alignment for a given scoring scheme, gap penalty *g*, and temporal penalty *T*_*p*_. Notwithstanding, alignments can drastically change depending on the choice of these parameters, and this is the reason why they should be carefully chosen.

The information of the retrieved alignments is summarized into an *N*×*N* similarity matrix *S*, where *N* is the number of patients in the data. In this matrix, the entry value (*i,j*) gives the alignment score of the *i*-th and *j*-th patients. Due to symmetry, only *N*×(*N*−1)/2 entries need to be computed.

### Distance matrix

Before using the agglomerative clustering algorithm, we need to convert the similarity matrix *S*, which was obtained in the previous step, into a distance matrix *D*. To this end, we take the symmetric value of each score and then we shift it by adding the maximum similarity score in matrix *S*. This shift is made in order to make all scores greater than or equal to zero. In summary, the distance matrix is computed as follows: *a*= max*i*<*jS*_*ij*_ with *i,j*=1,…,*N* and *D*=−*S*+*a*(**1**·**1**^*T*^) with $\mathbf {1} = \left (\begin {smallmatrix} 1 \\ \vphantom {\int \limits ^{x}}\smash {\vdots } \\ 1 \end {smallmatrix}\right) \in \mathbf {R}^{N}$.

### Clustering of temporal sequence alignments

The dissimilarity matrix obtained is then used to perform agglomerative clustering [16]. The resulting groups can be depicted in a dendrogram, a tree showing the order and distances of the merges performed during the clustering procedure. Five different linkage functions are used, namely, single, complete, average, centroid, and Ward’s method. Since hierarchical clustering methods do not explicitly set the number of clusters, the AliClu additionally provides an automatic bootstrapping-based validation technique proposed by Mucha [17] that selects the best number according to several clustering indices. These indices include *Rand* [18], the *adjusted Rand* (AR) [19], *Fowlkes and Mallows* (FM) [20], *Jaccard*, and the *adjusted Wallace* (AW) [21].

The pseudo-code of the cluster and validation procedure is given in Algorithm 1. The inputs of the algorithm are the distance matrix *D* for the agglomerative clustering algorithm, the number of bootstrap samples *M*, the linkage criterion *L*, and the minimum *K*_min_ and the maximum *K*_max_ numbers of clusters to be analysed. The output is the statistics of all the clustering indices described above, namely, the medians, means, and variances for all the bootstrap samples, which are calculated for each analysed number of clusters (between *K*_min_ and *K*_max_).


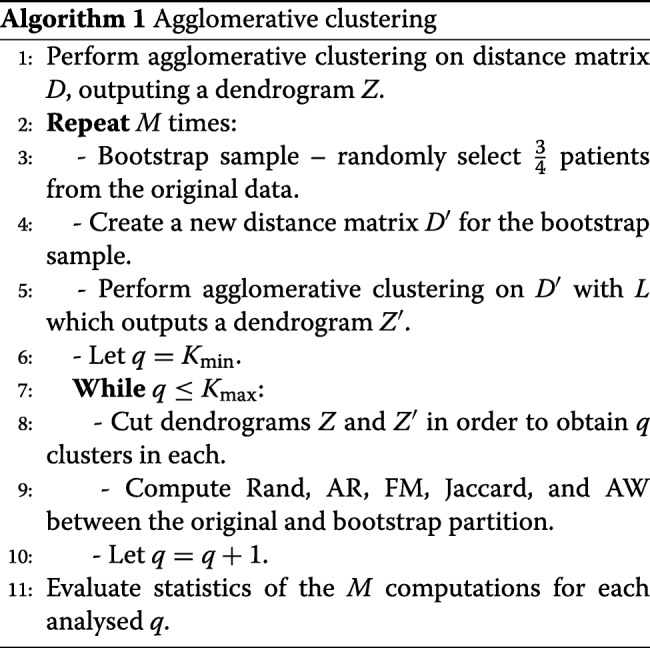


The algorithm begins by performing agglomerative clustering on distance matrix *D* in Step 1. Then, an outer loop starts in Step 2, corresponding to a bootstrapping procedure. From Steps 3 to 5, a bootstrapped sample is generated, and agglomerative clustering is performed on it. Then, an inner loop computes the clustering indices between the clustering of the original patients and the clustering of the bootstrapped sample (Steps 6-10). In Step 8, the obtained dendrograms *Z* and *Z*^′^ are cut to retrieve *q* clusters (in each), where *K*_min_≤*q*≤*K*_max_. After running the outer loop *M* times, the statistics of the clustering indices are computed (Step 11).

The output of Algorithm 1 helps to select the best number of clusters in the data, herein *k*. The right candidate is the one that yields the higher number of maximum average values over the clustering indices. To corroborate the previous guess, the standard deviation of the clustering indices for each *k* can be taken into account. The choice of *k* can be automatic or semi-automatic. In this latter case, the results composed by dendrograms, the averages and the standard deviations of the obtained clustering indices are given to the user for manual inspection and further selection.

After obtaining the best number of clusters *k* according to these criteria, the stability of each individual cluster is then assessed in Algorithm 2, again via the bootstrapping approach [17]. The inputs of this algorithm are the number of clusters *k*, the clusters themselves {*A*_1_,…,*A*_*k*_}, the linkage criterion *L*, and the number of bootstrapped samples *M*. The output is the stability measures of the obtained clusters, which are assessed by the criteria described as follows.


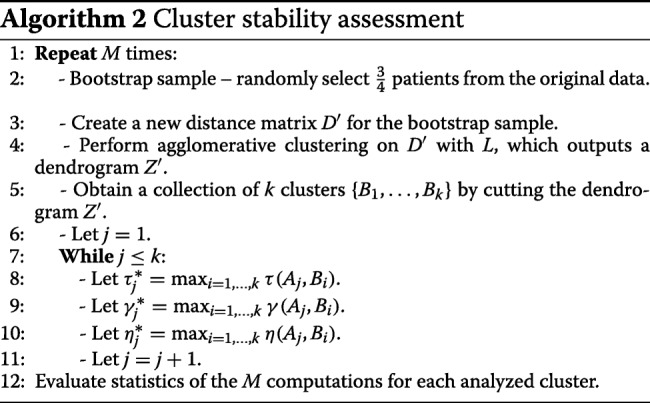


The algorithm starts with resampling. For each bootstrapped sample, a dendrogram *Z*^′^ is obtained by performing agglomerative clustering on the sample (Steps 2-4). Then, a collection of *k* clusters {*B*_1_,…,*B*_*k*_} is obtained by cutting the dendrogram *Z*^′^ (Step 5). From Steps 6 to 11, as proposed by Mucha [17], three different measures are computed for each cluster *A*_*j*_,1≤*j*≤*k*, namely, $\tau _{j}^{\ast }$ (the Jaccard index), $\gamma _{j}^{\ast }$ (the recovery rate) and $\eta _{j}^{\ast }$ (the Dice coefficient). These indices provide a measure of the similarity between cluster *A*_*j*_ and its most similar cluster in {*B*_1_,…,*B*_*k*_}. Finally, in Step 12, the stability of the retrieved clusters is assessed by computing the average values of $\tau _{j}^{\ast }, \gamma _{j}^{\ast }$ and $\eta _{j}^{\ast }$, and by analysing the corresponding standard deviations.

As discussed in [17], it is difficult to set an appropriate threshold that denotes that a cluster is stable. Therefore, we followed the rule of thumb and considered stable clusters as the ones that yield high average values (close to one) and low standard deviations for $\tau _{j}^{\ast }, \gamma _{j}^{\ast }$ and $\eta _{j}^{\ast }$.

Algorithm 3 presents the overall proposed method for obtaining clusters from PE sequences. Its inputs are the raw data, the scoring system *SS*, the temporal penalty *T*_*p*_, and the gap related parameters (*g*_min_,*g*_max_ and *g*_*istep*_) required by the TNW; the number of bootstrapped samples *M*, for Algorithm 1 and Algorithm 2; the linkage criterion *L*; and the minimum *K*_min_ and the maximum *K*_max_ numbers of clusters.


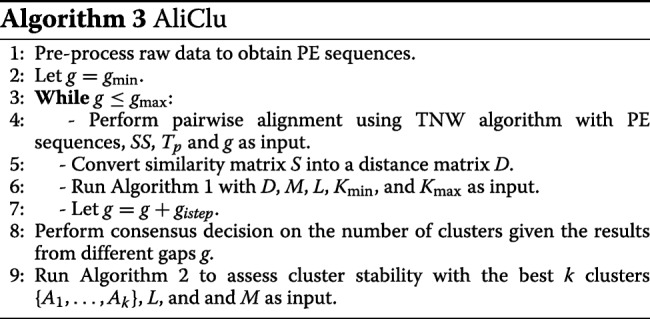


The initial step of the algorithm pre-processes the raw data to produce PE sequences (Step 1). The gap penalty of the TNW algorithm is then set to range from *g*_min_ to *g*_max_ at incremental steps of *g*_*istep*_ (Step 2 and Step 7). For each value of the gap penalty *g*, pairwise temporal alignment using the TNW is performed, which outputs a similarity matrix *S* (Step 4). Then, *S* is converted into a distance matrix *D* (Step 5). Clustering is then performed by running Algorithm 1 (Step 6).

When the cycle from Steps 3 to 7 ends, there are several results to explore: one for each of the number of clusters (*K*_min_,…, *K*_max_) and gap penalties (*g*_min_ to *g*_max_ with *g*_*istep*_). In Step 8, the final number of clusters *k* is obtained from these results. As stated before, if an automatic procedure is chosen, the final number of clusters *k* retrieved in this step is that which results in the most frequent higher average values for the clustering indices. In this case, the chosen gap penalty *g* is the one that yields the best average values for the clustering indices for the final number of clusters. In the semi-automatic option, the full results for different *k* and *g* – including the dendrograms, averages and standard deviations of the clustering indices – are provided to the user, which then determines the final number of clusters *k* and gap parameter *g* to be further used. In Step 9, the stability of the retrieved clusters is assessed by running Algorithm 2.

The run-time complexity of the TNW is *O*(*n*^2^), and that of agglomerative clustering is *O*(*N*^3^), where *n* is the length of the PE sequences and *N* is the number of patients in the data. Moreover, computing the cluster stability in Algorithm 2 for Steps 6–11 takes $O(K_{\max }^{2}\times N)$. Therefore, the AliClu algorithm takes

$$O(\Delta G\times n^{2} + \Delta G\times M\times \Delta K\times N^{3} + M\times K_{\max}^{2} \times N)$$

time, where $\Delta G=\left \lceil \frac { g_{\max }-g_{\min }+1}{g_{istep}}\right \rceil $ is the number of gaps analysed (*g*_min_ to *g*_max_ with *g*_*istep*_), *M* is the number of bootstrapped samples, and *ΔK*=*K*_max_−*K*_min_+1 is the number of clusters considered (from *K*_min_ to *K*_max_).

## Results

### Synthetic datasets

We first evaluate the AliClu using synthetic datasets, which provides a proof of concept in a controlled scenario where the true cluster labels are known a priori and makes it easy to determine the merits of the method. The synthetic datasets consisted of temporal sequences generated by *continuous-time Markov chains* in a variety of parameter settings.

We concluded that the AliClu successfully found the correct clusters in more than 80*%* of the cases for datasets containing two well-separated clusters. Moreover, the linkage method that produced the best results for the agglomerative clustering was Ward’s method; thus, it was adopted in the remaining experiments. The complete study of the AliClu behaviour on each of the synthetic problems is available in the Additional file 1, along with all the details regarding the sequence generation and clustering evaluation.

### The Reuma.pt database

We then applied the AliClu to biologic therapy switching for *rheumatoid arthritis* (RA) patients in a real-life longitudinal cohort – the Reuma.pt database [11].

Reuma.pt [11] is a Portuguese nationwide database developed by the Portuguese Society of Rheumatology. It stores the EMRs of rheumatoid patients as structured and narrative data with the goal of monitoring the disease’s progression and assuring treatment effectiveness and safety. In this study, we focus on patients with *rheumatoid arthritis* (RA) being treated with biologic therapies at one centre. The retrieved data include 426 patients diagnosed with RA who followed-up regularly more or less every three to six months, which resulted in a total of 9305 medical appointments.

The RA is an immunomediated inflammatory rheumatic disease that causes pain and swelling in the wrists and small joints of the hands and feet. RA treatments can mitigate these symptoms, prevent joint damage, and provide a better quality of life to the patients. Traditional therapies consist of using conventional *disease-modifying antirheumatic drugs* (DMARDs), which are used as a monotherapy or in combinations. When patients fail to respond to conventional DMARDs, modern biologic therapies are tried. Unlike conventional DMARDs, biologic ones are made using biotechnology. Biologics are genetically engineered to act as natural proteins in the human immune system.

The goal of RA treatment is to induce the disease’s remission by controlling the inflammation. This approach would relieve the symptoms, prevent joint and organ damage, improve physical functioning and overall well-being, and reduce long-term complications. It is crucial to identify the most effective RA treatments early in the disease’s progression. In this regard, we used the AliClu to analyse biologic therapy switching, where PE sequences are built by interspersing biologic drugs that are coded as letters and include their durations. The optimized clusters allow for the stratification of RA patients based on their temporal therapy profiles and identification of common features of these groups. Patients starting new biologic therapies can then benefit from these insights.

### Clustering of biologic therapy switches

**Fig. 2:**
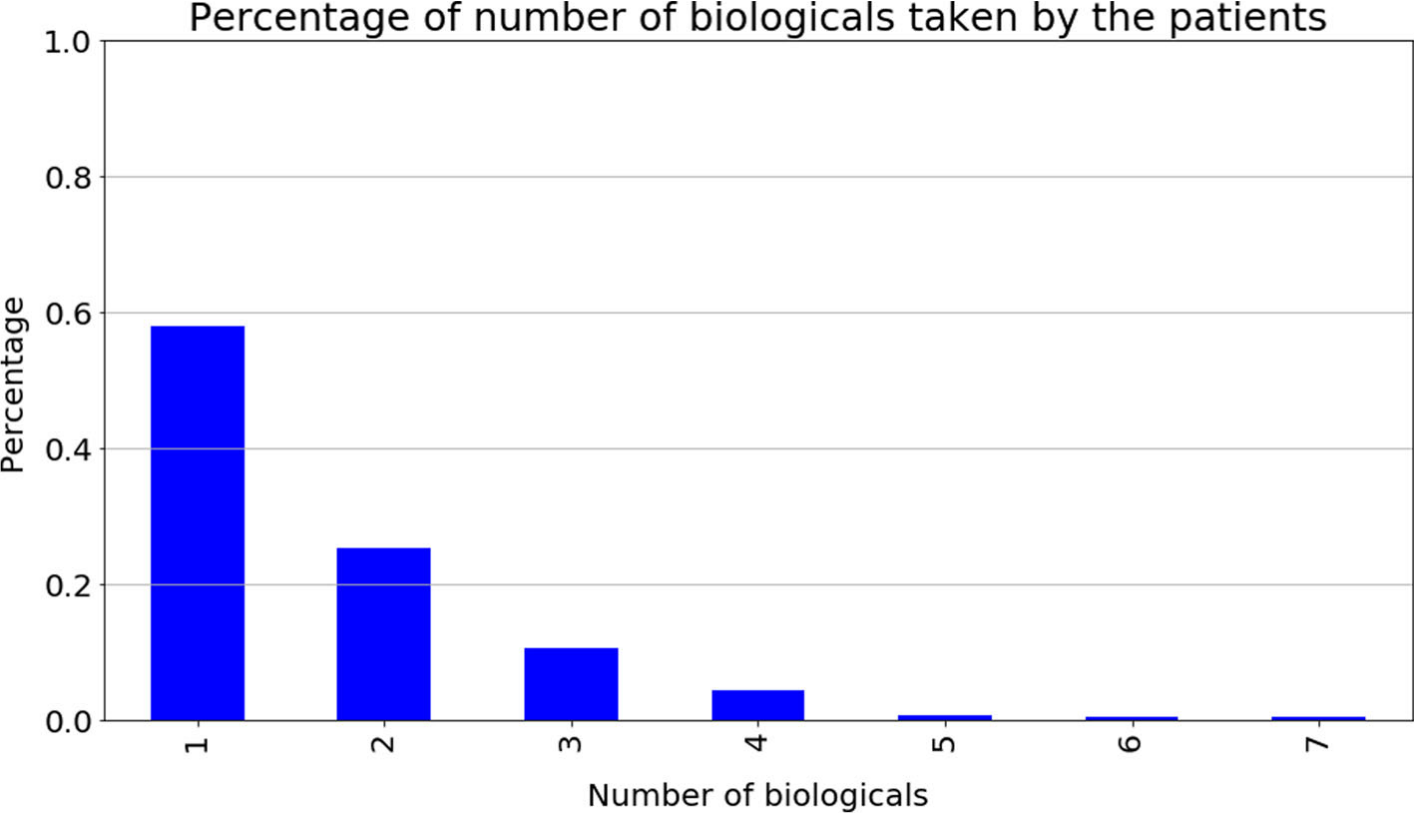
Percentage of biologic drugs taken by Rheumatoid Arthritis (RA) patients. Almost 60% of the patients only had one biologic drug. Patients that have taken more than five biologic drugs are rare; three patients have taken five, two patients have taken six, and other two seven biologic drugs

Data of the 426 RA patients concerning biologic therapy switches from the Reuma.pt database were preprocessed to build the PE sequences. Figure 2 presents the statistics regarding the number of biologic drugs taken by patients. Almost 60% of the patients had only one biologic drug recorded (no switches). Patients who have taken five or more drugs are rare: three patients have taken five, two have taken six, and two have taken seven different treatments. We stress that when switching therapies, a patient never goes back to taking the previous biologic drug.

For this particular dataset, the following drugs were as follows: A – etanercept, B – infliximab, C – rituximab, D – adalimumab, E – anacinra, F – abatacept, G – tocilizumab, and H – golimumab. These drugs correspond to distinct active therapeutic principles and are prescribed in different stages of the disease.

Having the PE sequences, Algorithm 3 is run with *K*_max_=30, and all other input parameters are set to their default values. The scoring system is 1 for a match and −1.1 for a mismatch of the drug representation, the temporal penalty is *T*_*p*_=0.25, and the number of bootstrapped samples is *M*=1000. Moreover, in this experiment, the AliClu is used in a semi-automatic manner (Step 12 of Algorithm 1 and Step 8 of Algorithm 3 are subject to user input).

We concluded that Ward’s linkage leads to superior results in terms of the clustering indices and clinical information, and a gap penalty of *g*=0.7 and a temporal penalty of *T*_*p*_=0.25 correspond to balanced choices with respect to the other input parameters. It is noteworthy that these choices are data dependent and provide a proof-of-concept of the principle since a full analysis and optimization of the clustering parameters would be out of the scope of the present work.

The running time recorded for this final setting was approximately 1 hour by using a machine with a 2.6 GHz Intel Core i7 processor and 16 GB of 2400 MHz DDR4 memory. This time corresponds to approximately 3.8 seconds for each gap and replicate analysed for the full range of cluster numbers.

**Fig. 3:**
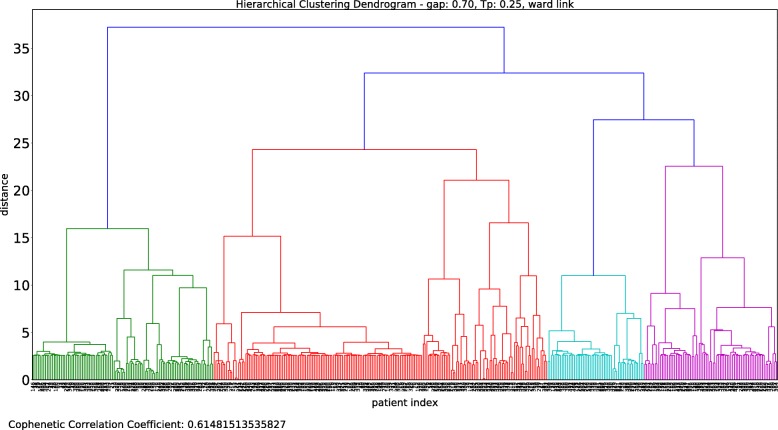
Dendrogram of the agglomerative hierarchical clustering of Rheumatoid Arthritis (RA) patients. Dendrogram of Ward’s method hierarchical clustering with gap penalty *g*=0.7 and temporal penalty *T*_*p*_=0.25. Twenty five clusters were selected based on the analysis of the clustering indices and clinical interpretation

Figure 3 shows the dendrogram obtained when using this parameter set, i.e., *g*=0.7 and a temporal penalty of *T*_*p*_=0.25. The averages of the five clustering indices obtained with Algorithm 1 are presented in Table 1.

**Table 1:** Average values of five clustering indices for the dendrogram of Fig. 3

*k*	Rand	AR	FM	Jaccard	AW
2	0.876	0.744	0.897	0.827	0.704
3	0.852	0.675	0.789	0.658	0.661
4	0.872	0.689	0.780	0.644	0.644
5	0.897	0.705	0.773	0.632	0.759
6	0.920	0.751	0.802	0.672	0.768
7	0.935	0.780	0.820	0.699	0.771
8	0.931	0.753	0.796	0.662	0.700
9	0.950	0.801	0.830	0.712	0.782
10	0.966	0.855	0.875	0.779	0.861
11	0.969	0.863	0.881	0.789	0.857
12	0.973	0.876	0.892	0.805	0.878
13	0.975	0.883	0.897	0.814	0.883
14	0.979	0.897	0.909	0.833	0.914
15	0.982	0.910	0.920	0.852	0.917
16	0.985	0.925	0.933	0.875	0.931
17	0.987	0.932	0.940	0.887	0.937
18	0.988	0.936	0.943	0.893	0.939
19	0.989	0.940	0.946	0.899	0.944
20	0.988	0.937	0.943	0.893	0.933
21	0.989	0.938	0.945	0.895	0.939
22	0.990	0.942	0.948	0.901	0.940
23	0.991	0.946	0.951	0.907	0.961
24	0.992	0.953	0.958	0.919	0.965
25	**0.993**	0.958	0.962	0.926	**0.966**
26	**0.993**	**0.959**	**0.963**	**0.929**	0.964
27	**0.993**	0.958	0.962	0.928	0.960
28	0.992	0.955	0.959	0.923	0.952
29	0.992	0.952	0.957	0.920	0.945
30	0.991	0.940	0.947	0.903	0.924

Three of the measures, namely, the AR, FM, and Jaccard, indicate the existence of 26 clusters; the AW indicates that *k*=25, and the AR indicates that *k*=25, 26 and 27. In this case, not all averages point to the same number of clusters *k*; therefore, a more careful and refined analysis is required.

**Fig. 4:**
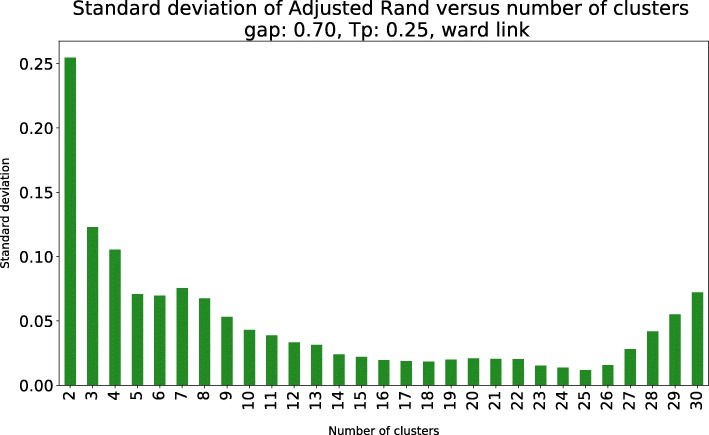
Standard deviation of Adjusted Rand (AR) versus the number of clusters. Standard deviation of AR versus number of clusters for dendrogram in Fig. 3. There is a downward trend of the standard deviation when increasing the number of clusters. The minimum value is attained with 25 clusters

We complemented this analysis with the standard deviation of the AR, which is presented in Fig. 4. The minimum standard deviation of the AR is achieved for *k*=25, which, combined with the information provided in Table 1 and Fig. 4, leads to the selection of 25 clusters.

The stability of the 25 clusters was then assessed through the medians, averages and standard deviations of *η*^∗^,*τ*^∗^ and *γ*^∗^ (Table 2). As expected, the three statistic values of *η*^∗^ are always smaller than those of *τ*^∗^ and *γ*^∗^. For some clusters, the medians and averages of the three measures are not as high as is desirable to consider the clusters stable. Moreover, the medians and averages of *τ*^∗^ and *γ*^∗^ are not the same in all clusters. Notwithstanding, in clusters 20,21,22,23,24, and 25 (also those with more observations), those values are the same, and they are high enough to be considered stable.

**Table 2:** Stability of the 25 clusters for Ward’s method, *g*=0.7, and *T*_*p*_=0.25

Cluster Nb.	*τ*^∗^	*η*^∗^	*γ*^∗^	*τ*^∗^	*η*^∗^	*γ*^∗^	*τ*^∗^	*η*^∗^	*γ*^∗^
(# patients)	median	median	median	average	average	average	std	std	std
1 (4)	0.475	0.298	0.625	0.475	0.298	0.625	0.389	0.185	0.177
2 (4)	0.750	0.429	0.750	0.750	0.429	0.750	0.000	0.000	0.000
3 (5)	0.083	0.077	0.200	0.083	0.077	0.200	0.000	0.000	0.000
4 (5)	0.400	0.271	0.600	0.400	0.271	0.600	0.283	0.147	0.000
5 (5)	0.275	0.215	0.500	0.275	0.215	0.500	0.035	0.022	0.141
6 (6)	0.833	0.455	0.833	0.833	0.455	0.833	0.000	0.000	0.000
7 (7)	0.741	0.423	0.786	0.741	0.423	0.786	0.164	0.054	0.101
8 (7)	0.307	0.233	0.500	0.307	0.233	0.500	0.080	0.047	0.101
9 (7)	0.643	0.390	0.643	0.643	0.390	0.643	0.101	0.037	0.101
10 (8)	0.688	0.407	0.688	0.688	0.407	0.688	0.088	0.031	0.088
11 (9)	0.542	0.347	0.611	0.542	0.347	0.611	0.177	0.075	0.079
12 (9)	0.389	0.269	0.444	0.389	0.269	0.444	0.236	0.124	0.157
13 (10)	0.352	0.256	0.400	0.352	0.256	0.400	0.145	0.080	0.141
14 (10)	0.489	0.311	0.550	0.489	0.311	0.550	0.337	0.156	0.354
15 (13)	0.513	0.330	0.577	0.513	0.330	0.577	0.254	0.112	0.163
16 (13)	0.472	0.321	0.577	0.472	0.321	0.577	0.039	0.018	0.054
17 (14)	0.571	0.358	0.571	0.571	0.358	0.571	0.202	0.082	0.202
18 (16)	0.719	0.416	0.719	0.719	0.416	0.719	0.133	0.045	0.133
19 (17)	0.309	0.235	0.353	0.309	0.235	0.353	0.084	0.049	0.083
20 (19)	0.716	0.416	0.737	0.716	0.416	0.737	0.119	0.041	0.149
21 (20)	0.791	0.440	0.825	0.791	0.440	0.825	0.154	0.048	0.106
22 (32)	0.696	0.410	0.719	0.696	0.410	0.719	0.056	0.019	0.088
23 (37)	0.791	0.441	0.811	0.791	0.441	0.811	0.104	0.032	0.076
24 (46)	0.728	0.420	0.728	0.728	0.420	0.728	0.108	0.036	0.108
25 (101)	0.777	0.437	0.777	0.777	0.437	0.777	0.007	0.002	0.007

### Clusters visualization

Visualization is an essential task in any clustering process since it provides an intuitive way to validate clusters. Due to the characteristics of the clustered PE sequences, we propose a graph representation that summarizes the information regarding the sequences that belong to a given cluster. Therein, each node represents a biologic drug symbol (“A” to “H”, and “Z” described above), and each edge represents a therapy switch (from one biologic drug to another). A special symbol “Z” marks the end of the sequence, signalling that from that point on there is no information regarding the therapy’s success or failure. The value on an edge is the median of the times between the corresponding drug switches in that cluster.

The colour of an edge represents the transition probability from one biologic drug to another. This probability is computed by counting the number of times a switch occurs divided by the total number of transitions in that cluster. A grey scale is used for the edges in this regard. A darker edge means that the switches between the linked biologic drugs frequently occurred in that cluster.

**Fig. 5:**
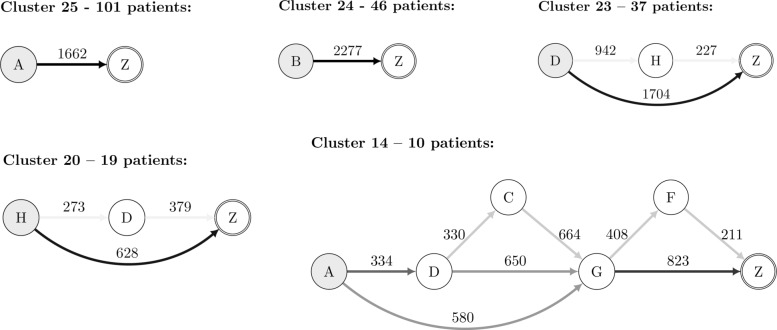
Cluster Visualization. Graph representation of selected clusters based on stability measures and clinical interpretation. Drug codes: A - Etanercept; B - Infliximab; C - Rituximab; D - Adalimumab; E - Anacinra; F - Abatacept; G - Tocilizumab; H - Golimumab. Z - Follow-up/end

The clusters with higher stability correspond to easily interpretable therapy profiles, including monotherapies (no switches). For example, these include clusters with only etanercept (A; Cluster 25 – 101 patients), only infliximab (B; Cluster 24 – 46 patients), or minor or no switches for the majority of the patients in that group. For example, cluster with adalimumab (D; Cluster 23 – 37 patients) where some patients switch to golimumab (H), and vice-versa (Cluster 20 – 19 These clusters are represented in Fig. 5. Less stable clusters may also provide relevant clinical information regarding the longitudinal profile of the therapy. For example, Cluster 14 (with 10 patients), defines a more elaborate structure of therapy switches, which corresponds to a more complex medical interpretation. Patients started with a TNF inhibitor agent (etanercept, A). If the patient’s therapy failed (secondary failure) after some time, then the patient can be switched to a new TNF inhibitor (adalimumab, D). After two TNF inhibitor agents failed, the patients were switched to another class of drugs. The next drug can be either a B cell antibody (rituximab, C) or an IL-6 inhibitor (tocilizumab, G). Sometimes, patients do not respond at all to the first TNF inhibitor agent (primary failure) or they can develop severe adverse reactions. In those cases, the rheumatologist can decide to go directly from etanercept (A) to tocilizumab (G) and switch the drug class earlier. This example shows a direct meaningful interpretation of the obtained clusters from a medical point of view and highlights the advantages of patient stratification using longitudinal data.

## Conclusions

We propose the AliClu, a method that combines temporal sequence alignment and agglomerative hierarchical clustering to find groups in longitudinal data containing sequences of symbols and numeric values. The AliClu includes a clustering validation strategy based on bootstrapping and uses several clustering indices, such as the (adjusted) Rand, Fowlkes–Mallows, Jaccard, and adjusted Wallace, to choose the best number of groups to consider for each particular dataset. The stability of the obtained clusters is then assessed through resampling and by using the Jaccard index, the recovery rate, and the Dice indices coefficient. The AliClu can either be run entirely automatically or in a semi-automatic way, which requires user input regarding the chosen parameters. The final clusters are depicted in graphs where each node represents a symbol, each edge (a state switch) has one number corresponding to the median time, and the weight represents the estimated conditional probability of switching.

The AliClu was tested using synthetic data generated with continuous-time Markov chain models, which makes it possible to separate the sequences generated with different parameters. The AliClu was run using the Portuguese Rheumatic Diseases Register (Reuma.pt), the national database for all the rheumatic patients treated with biologic agents. In particular, the rheumatoid arthritis (RA) patients’ therapy information, including the sequence of drugs taken and their durations, was used as the input. The procedure allowed us to stratify RA patients in a clinically relevant way by creating groups of similar treatment profiles. The clusters obtained depict the treatment switches between different drugs, their median duration times and their probabilities.

The AliClu provides a strategy setting, validation, and visualization procedure for the automatic clustering of temporal sequence data, and it has promising applications for patient stratification using electronic medical record (EMR) data.

## Availability and requirements

**Project name**: AliClu

**Project home page**: https://github.com/sysbiomed/AliClu

**Operating system(s)**: Platform independent

**Programming language**: Python

**Other requirements**: Python3 (in Linux or Windows) and Anaconda (in Mac OS)

**License**: Free

**Any restrictions to use by non-academics**: None

## Supplementary information

**Additional file 1** Supplementary Information

## Data Availability

AliClu is available at https://github.com/sysbiomed/AliClu. Data from Reuma.pt are not publicly available. Synthetic data is provided along with AliClu to ease its use.

## Abbreviations

AR: adjusted Rand
AW: adjusted Wallace
DMARD: disease-modifying antirheumatic drugs EMR: Electronic Medical Records
FM: Fowlkes and Mallows
PE: prefix-encoded
RA: rheumatoid arthritis
TNW: Temporal Needleman-Wunsch
