# Supplementary material for: AliClu - Temporal sequence alignment for clustering longitudinal clinical data
Source: BMC Med Inform Decis Mak. 2019 Dec 30;19:289. doi: 10.1186/s12911-019-1013-7 (PMC6938005; doi:10.1186/s12911-019-1013-7)
Supplement: Supplementary file 1 — Additional file 1 Supplementary Information [file 12911_2019_1013_MOESM1_ESM.pdf]

Additional File

AliClu – Temporal sequence alignment for clustering longitudinal clinical data

Kishan Rama, Helena Canhão, Alexandra M. Carvalho and Susana Vinga

Contents

1 Introduction **1**

2 Pre-Processing **1**

2.1 Pre-Processing I: time is a number 2

2.2 Pre-Processing II: time is a date 3

3 Pairwise alignment **4**

3.1 Needleman-Wunsch algorithm 4

3.2 Temporal Needleman-Wunsch algorithm 8

4 Synthetic results **10**

4.1 Temporal sequence generation 11

4.2 Two clusters from the same two-state transition diagram 12

4.3 Four clusters from different transition diagrams 13

1 Introduction

In this Additional File we present all the details required for pre-processing data, from panel to prefix-encoded sequences, needed by the TNW algorithm. Afterwards, we present the temporal Needleman-Wunsch algorithm and results of the AliClu algorithm on synthetic data.

2 Pre-Processing

Longitudinal data is usually available in a panel format. Therein, each row or line corresponds to a medical appointment of a certain patient; the columns contain the several

features measured during the medical appointments.

In this work, we consider that each patient experiences a sequence of events spaced in time. Let A and B be events of interest for a given patient, with time-distance t between them; a *prefix-encoded* (PE) sequence for that patient is defined as $0.A, t.B$. Three variables are required: *id_patient*, *event* and *time*. The *id_patient* refers to the unique identifier of the patient, *event* is the observed feature and *time* is the variable that provides the temporal information. For each patient, a temporal sequences is built as follows. The observed feature *event* is always categorical. If a numerical numerical one is desired a discretization step must be done before proceeding. In addition, the variable *time* can appear formatted as a date or just as a number in any time unit (seconds, minutes, days, etc.). Depending on the type of the *time* variable we can have two different pre-processing steps, as described in the next sections.

2.1 Pre-Processing I: time is a number

In this case the *time* variable is just a number. An example of raw data that needs this type of pre-processing is presented in Table 1. Each row shows an event experienced by a

id_patient	*event*	*time*
12	2	73
12	3	45
12	4	91
15	2	135
20	0	NA
20	1	115

Table 1: Example of raw data for pre-processing I. The *event* variable is categorical and *time* is a number representing the time, in days, between two consecutive events.

certain patient at a medical appointment. The *time* variable correspond to the duration of the associated *event*. Missing values are represented as *not available* (NA). Three patients are presented: patient 12 experienced 3 events, patient 15 experience only one, and patient 20 experienced two events.

For each patient with id *id_patient*, the pre-processing steps are as follows:

1. Eliminate rows with NA values.

2. If *event* is a categorical variable represented with a number, convert this number into a letter in alphabetic order; e.g. $1 \rightarrow$ A, $2 \rightarrow$ B, etc. This is done to distinguish

transition times (*time*) from events (*event*). If *event* is already a letter, this step is skipped.

3. Create the prefix-encoded temporal sequences.

For illustration purposes, the temporal sequences obtained with the data presented in Table 1 are presented in Table 2.

id_patient	PE temporal sequence
12	0.B,73.C,45.D,91.Z
15	0.B,135.Z
20	0.A,115.Z

Table 2: Prefix-encoded temporal sequences for patients 12, 15 and 20 in Table 1.

We note that event Z is artificially introduced to mark the end of a sequence, hence, the last observed event of a patient is given by the penultimate event in the sequence.

2.2 Pre-Processing II: time is a date

Herein, *time* is formatted as a date. An example of raw data that needs this type of pre-processing is presented in Table 3. In this example the observed variable *event* is again categorical, but this time already represented as a letter. In this case, pre-processing raw data requires one additional step (when compared with Pre-processing I) to compute the time interval between two consecutive events.

For each patient with id *id_patient*, the main pre-processing steps are:

1. Eliminate rows with NA values.

2. If *event* is a categorical variable represented with a number, convert this number into a letter in alphabetic order; e.g. 1 $\rightarrow$ A, 2 $\rightarrow$ B, etc. If *event* is already a letter, skip this step.

3. Compute the time elapsed between two consecutive events and associate it with the corresponding row.

4. Create the prefix-encoded temporal sequences.

The temporal sequences obtained with data in Table 3 are presented in Table 4. Therein, for instance, patient with id number 12 has a transition between event A and B of 12 days. Note, however, that it is not possible to build a temporal sequence for patient 15 from Table 3, since there is no information available on the *event* variable.

id_patient	*event*	*time*
12	A	03/10/2017
12	B	15/10/2017
12	A	22/10/2017
12	NA	6/11/2017
12	NA	18/11/2017
12	C	08/12/2017
15	NA	14/03/2017
20	B	10/01/2018
20	C	20/01/2018
20	D	02/02/2018

Table 3: Example of raw data for pre-processing II. The *event* variable is categorical and *time* is in date format representing the date of the medical appointments.

id_patient	PE temporal sequence
12	0.A,12.B,7.A,17.C
20	0.B,10.C,13.D

Table 4: Prefix-encoded temporal sequences for patients 12 and 20 built from data in Table 3.

After the pre-processing step, all patients are fully characterized by the temporal sequences. These sequences provide the clinical history of the patient regarding a specific observed variable *event*. Data in this format is the input for the Temporal Needleman-Wunsch (TNW) algorithm.

3 Pairwise alignment

Herein, we present the Needleman-Wunsch (NW) algorithm and then its temporal extension.

3.1 Needleman-Wunsch algorithm

The NW algorithm was developed by Saul B. Needleman and Christian D. Wunsch and published in 1970. It is proven to find the optimal alignment between two sequences. It is based on dynamic programming where the basic idea is to solve the problem by dividing it

into smaller ones; solve the smaller problems optimally and then use the sub-solutions to construct an optimal solution for the original problem.

For a pair of sequences,

$$X = x_1, ..., x_m \text{ and } Y = y_1, ..., y_n,$$

where x_i and y_j, for $i \in [1, m]$ and $j \in [1, n]$, are the symbols of sequences X and Y with size m and n, respectively. The algorithm uses two matrices – score matrix H and traceback matrix T – that considers all possible pairs of symbols in the two sequences to build the alignment. The NW algorithm consists of three steps:

1. Initialisation of the score matrix H:

$$H_{r,0} = -rg; H_{0,c} = -cg \quad \forall_{r\in[0,m],c\in[0,n]}. \tag{1}$$

2. Calculation of the scores and filling the traceback matrix T. The remaining entries of the score matrix H are defined as:

$$H_{i,j} = \max \begin{cases} H_{i-1,j-1} + S(x_i, y_j) \\ H_{i-1,j} - g \\ H_{i,j-1} - g \end{cases} , \quad \forall_{i\in[1,m],j\in[1,n]}. \tag{2}$$

3. Deducing the alignment from the traceback matrix T.

In Equations (1) and (2), $S(x_i, y_j)$ is a user-defined *scoring schema* that measures the similarity between symbols x_i and y_j in the sequences. Moreover, g is a user-defined constant, called the *gap penalty*, that allows to penalize the alignment score whenever a gap is inserted.

Example To illustrate the steps of the algorithm consider two sequences given by:

$$X = SEND \qquad Y = AND.$$

We start by defining a scoring schema $S(x, y)$, presented in Figure 1. If two symbols x_i and y_j of our sequences are equal then the matching score is 1 ($S(x_i, y_j) = 1$) otherwise we have a mismatch and the score is either -1 ($S(x_i, y_j) = -1$) or -2 ($S(x_i, y_j) = -2$). The gap penalty is assumed to be 2 ($g = 2$).

Now we follow the three steps described above:

	**A**	**D**	**E**	**N**	**S**
**A**	1	-1	-2	-1	-1
**D**	-1	1	-1	-1	-1
**E**	-2	-1	1	-1	-1
**N**	-1	-1	-1	1	-1
**S**	-1	-1	-1	-1	1

Figure 1: User-defined scoring schema S.

1. **Initialisation step**: The score matrix H is initialized with $m + 1$ rows and $n + 1$ columns where m and n are the size of sequences X and Y, respectively. The extra row and column is given for alignments with gaps. With Equation (1) we fill the first row and first column (c.f. Figure 2). The traceback matrix T is initialised according to Figure 3.

		**S**	**E**	**N**	**D**
	0	-2	-4	-6	-8
**A**	-2				
**N**	-4				
**D**	-6				

Figure 2: Initialisation of score matrix H.

		**S**	**E**	**N**	**D**
	done	left	left	left	left
**A**	up				
**N**	up				
**D**	up				

Figure 3: Initialisation of traceback matrix T.

2. **Matrix H fill step**: The remaining entries of matrix H are filled by row or column starting at $H_{1,1}$ by using Equation (2). At each position i, j the score is computed taking into account the values at three other positions at the immediately adjacent northwest diagonal, up and left cells. In Figure 4 (left side) is presented a pictorial representation of this computation for the first entry $(1, 1)$.

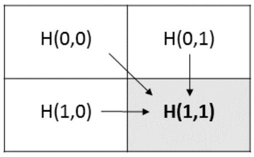

$$v_{diag} = H_{0,0} + S(A, S) = 0 + (-1) = -1$$
$$v_{up} = H_{0,1} - g = -2 - 2 = -4$$
$$v_{left} = H_{1,0} - g = -2 - 2 = -4$$

Figure 4: Pictorial representation of $H_{1,1}$ entry computation.

The value of $H_{1,1}$ is the maximum of the three values v_{diag}, v_{up} and v_{left} presented in Figure 4 (right side). Hence, $H_{1,1} = v_{diag} = -1$ and the corresponding traceback matrix cell is filled with "diag" (Figure 5). If instead of v_{diag} the maximum value

was v_{up} or v_{left} then the corresponding traceback matrix positions would be 'up' and 'left', respectively.

	**S**	**E**	**N**	**D**
0	-2	-4	-6	-8
**A** -2	**-1**			
**N** -4				
**D** -6				

		**S**	**E**	**N**	**D**
	done	left	left	left	left
**A**	up	**diag**			
**N**	up				
**D**	up				

Figure 5: Filling $H_{1,1}$ (left) and $T_{1,1}$ (right).

The same procedure is followed to compute all the other cells. The final score H and traceback T matrices are presented in Figure 6. The final score of the alignment is given by the last filled entry, corresponding in this example to the entry $H_{3,4} = -1$.

		**S**	**E**	**N**	**D**
	0	-2	-4	-6	-8
**A**	-2	1	-3	-5	-7
**N**	-4	-3	-2	-2	-4
**D**	-6	-5	-4	-3	-1

		**S**	**E**	**N**	**D**
	done	left	left	left	left
**A**	up	diag	left	left	left
**N**	up	diag	diag	diag	left
**D**	up	diag	diag	diag	diag

Figure 6: Final score (left) and traceback (right) matrices.

3. **Traceback step**: The alignment between the two sequences can now be deduced from the traceback matrix. The traceback procedure begins with the last filled entry (bottom right cell) and ends at the first matrix position (top left cell – "done"). At each position we move according to the value stored in it, i.e., the three possible moves are diagonal (northwest direction), up and left. In Figure 7 is shown the traceback that was performed on the matrix T with the alignments (numbered from 1 to 4) being done at each move.

   In the first move we look at the value in the bottom right cell that is "diag", which means that the pair "DD" of letters corresponding to the two sequences are aligned. Then we move diagonally from the position (3,4) to the position (2,3). The latter cell also stores the value "diag", hence, the same procedure applies here and the the pair "NN" is aligned. In the third step the cell (1,2) has the value "left". This means that a gap is introduced in the left sequence, i.e., the letter "E" from sequence "SEND" is aligned with a gap and the letter "A" from sequence "AND" will look for other

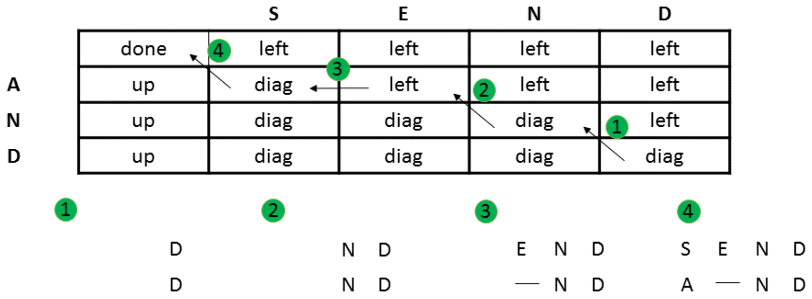

Figure 7: Traceback procedure using traceback matrix.

alignments while we traceback. Finnaly, in step 4 we encounter again the value "diag" at cell (1,1) and letter "A" is aligned with "S" concluding the alignment procedure.

In summary, the values stored in the traceback matrix indicate the alignment: "diag", where the letters from two sequences are aligned; "left", where a gap is introduced in the left sequence; "up", where a gap is introduce in the top sequence.

3.2 Temporal Needleman-Wunsch algorithm

The temporal Needleman-Wunsch (TNW) algorithm, proposed by Haider Syed and Amar K. Das, is a modified version of the NW method that incorporates the transition times between elements of a sequence. Therefore, it can be used for pattern discovery and matching sequences where time between events is relevant. The authors used the proposed method for autonomous chemotherapy-protocol recognition where patients treatment histories are aligned with standard recommended protocols to understand which protocol a patient is following.

The TNW algorithm can compute the temporal penalties with the total transition time between consecutive match pairs, by using PE sequences and two auxiliary matrices TR and TC that accumulate these transition times for events that align with gaps associated with the first X and second sequence Y, given by

$$X = 0.x_1, t_{x_1}.x_2, \dots, t_{x_{m-1}}.x_m \text{ and } Y = 0.y_1, t_{y_1}.y_2, \dots, t_{y_{n-1}}, \dots, y_n.$$

The score matrix H is calculated using:

$$H_{i,j} = \max \begin{cases} H_{i-1,j-1} + S(x_i, y_j) - f(t_{x_i} + TR_{i-1,j-1}, t_{y_j} + TC_{i-1,j-1}) \\ H_{i-1,j} - g \\ H_{i,j-1} - g \end{cases}, \quad (3)$$

for all $i \in [1, m]$ and $j \in [1, n]$, where TR and TC are given by

$$TR_{i,j} = \begin{cases} 0, & \text{if } T(i,j) = \text{“diag”} \\ TR_{i,j-1}, & \text{if } T(i,j) = \text{“left”} \\ TR_{i-1,j} + t_{xi}, & \text{if } T(i,j) = \text{“up”} \end{cases} \tag{4}$$

and

$$TC_{i,j} = \begin{cases} 0, & \text{if } T(i,j) = \text{“diag”} \\ TC_{i,j-1} + t_{yj}, & \text{if } T(i,j) = \text{“left”} \\ Tc_{i-1,j}, & \text{if } T(i,j) = \text{“up”}. \end{cases} \tag{5}$$

The only difference between Equations (2) and (3) is the introduction of a term $f(t_{x_i}, t_{y_j})$ that is a user defined temporal penalty function. The aim of this function is to reduce the similarity $S(x_i, y_j)$ by an amount that depends on t_{x_i} and t_{y_j}. In the original paper the following temporal penalty function was used:

$$f(t_{x_i}, t_{y_j}) = T_p \frac{|t_{x_i} - t_{y_j}|}{\max(t_{x_i}, t_{y_j})}, \tag{6}$$

where T_p is some user-defined constant, called *temporal penalty*, that will impose the maximum penalty on $S(x_i, x_j)$. This penalty function computes a percentage discrepancy between times t_i and t_j of two events x_i and x_j that are compared. If the events being compared have the same transition times associated to both of them that means that they are similar and the penalty function will be zero. But if the transition times are different then a penalty that depends on the times will be imposed. Indeed, instead of using the transition times of the match-pair alone ($-f(t_{x_i}, t_{y_j})$) this penalty function considers the total transition times between matched event pairs ($-f(t_{x_i} + TR_{i-1,j-1}, t_{y_j} + TC_{i-1,j-1})$ in Equation (3)) associated with the two sequence being alignment.

As an example, the result obtained by applying the TNW algorithm in the sequences

$$X = 0.A, t1.B, t2.C, t3.D \text{ and } Y = 0.A, t4.D$$

is shown in Figure 8 and Figure 9, where the resulting score H, traceback T, and the additional TR and TC matrices are presented. In this example the pre-defined scoring schema S consisted on scoring 1 matching events and -1.1 mismatching ones. The gap penalty was defined as 0.5. These scores were chosen in order reflect preference for aligning events with gaps instead of aligning mismatch events.

As already explained TR and TC matrices store values that represent the accumulated transition times for events that are aligned with gaps in the first sequence X and second sequence Y, respectively. This is clearly visible in Figure 9 where in the left side (TR

matrix) it is possible to see an accumulation of transition times (t_1, t_2, t_3) of sequence X as the row i increases; the same happens in the right side (TC matrix) but now column wise with only t_4 being accumulated. For example, the cell position $T_{2,0}$ indicates 'up' which means that there is no alignment; in this case, $TR_{2,0} = TR_{1,0} + t_{x_2} = 0 + t_1 = t1$ and $TC_{2,0} = TC_{1,0} = 0$. Because event B aligned to a gap we stored this transition time to use in a future calculation when some events align. In cases where we found a match, we add the diagonal TR and TC values to the transition times t_{x_i} and t_{y_j} of the events being compared. In the matrices positions $(4, 2)$ a match is discovered, hence, the temporal penalty is computed using $TR_{3,1} + t_{x_4} = t_1 + t_2 + t_3$ and $TC_{3,1} + t_{y_1} = 0 + t_4 = t_4$ which gives the function $-f(t_1+t_2+t_3, t_4)$ that is zero. Since we found a match pair the values for the corresponding cell positions of TR and TC are reset to zero. In this manner subsequent events that are aligned will use time information from the last pair of events that were matched.

		**0.A**	**t4.D**
	0	-0.5	-1
**0.A**	-0.5	1	0.5
**t1.B**	-1	0.5	0
**t2.C**	-1.5	0	-0.5
**t3.D**	-2	-0.5	1

		**0.A**	**t4.D**
	done	left	left
**0.A**	up	diag	left
**t1.B**	up	up	up
**t2.C**	up	up	left
**t3.D**	up	up	diag

Figure 8: H (left) and T (right) matrices.

		**0.A**	**t4.D**
	0	0	0
**0.A**	0	0	0
**t1.B**	t1	t1	t1
**t2.C**	t1+t2	t1+t2	t1+t2
**t3.D**	t1+t2+t3	t1+t2+t3	0

		**0.A**	**t4.D**
	0	0	t4
**0.A**	0	0	t4
**t1.B**	0	0	t4
**t2.C**	0	0	t4
**t3.D**	0	0	0

Figure 9: TR (left) and TC (right) matrices.

4 Synthetic results

In this section, we present results of the AliClu algorithm on synthetic data.

4.1 Temporal sequence generation

Synthetic data consist of temporal sequences generated by continuous-time Markov chains (CTMC). These type of models are adequate given the fact that they simulate very well both the transitions between discrete states and the corresponding times.

A CTMC is defined by two components: first, a discrete-time Markov chain, called the *jump chain*, whose set of states are denoted by E; second, a set of holding time parameters $\lambda = \{\lambda_i\}_{i \in E}$, where λ_i defines the average amount of time spent in the i-th state. A CTMC can be represented via a Q-matrix.

The matrix $Q = (q_{ij})_{i,j \in E}$, also referred to as the *generator matrix*, provides an alternative way of specifying a CTMC. This matrix has the following properties:

1. $q_{ii} \leq 0$ for all $i \in E$;

2. $q_{ij} \geq 0$ for all $i, j \in E$ such that $i \neq j$;

3. $\sum_{j \in E} q_{ij} = 0$ for all $i \in E$.

An adequate way to present the information contained in a Q-matrix is through a transition rate diagram. In this diagram, the values q_{ij} are shown on the edges, whereas the values of q_{ii} are not usually shown because they can be derived from the others.

For a given Q-matrix, the associated CTMC has a jump chain Π defined as follows:

$$\pi_{ij} = \begin{cases} \frac{q_{ij}}{q_i} & \text{if } q_i \neq 0 \text{ and } j \neq i \\ 0 & \text{if } (q_i \neq 0 \text{ and } j = i) \text{ or } (q_i = 0 \text{ and } j \neq 0) \\ 1 & \text{if } q_i = 0 \text{ and } j = i, \end{cases} \tag{7}$$

where $q_i = \sum_{i \neq j} q_{ij}$. Moreover, the holding parameters λ are such that $\lambda_i = q_i$, for all $i \in E$.

To generate temporal sequences, as described in Algorithm 1, an initial probability distribution vector α for the states is needed. The algorithm starts by initializing the CTMC with an initial state i being drawn from α. Then, in Step 2, the initial state i is converted into a letter, say I and the temporal sequence is initialized as $tseq =$'$0.I$'. Since, in our experiments, we never used more than five states, a simple conversion of the states into strings in alphabetical order is made, i.e a state $i = 1$ is converted to $I = A$, state $i = 2$ to $I = B$, etc. In Step 3, the time t of the jump of the CTMC to another state is simulated and then the new state j is drawn (Step 4). Again, after simulating the new state j a conversion to a latter J is made and the temporal sequence is updated as $tseq = tseq+$ '$,t.J$'. Finally, we consider the existence of an absorbing state, in order to mark the end of the sequences, hence, the simulation continues (Step 5) until an absorbing state is found.

Algorithm 1 Temporal sequence generation

1: Initialize the CTMC at $t = 0$ with initial state i drawn from the initial distribution α.

2: Convert the initial state i to a letter, say I, and initialize the temporal sequence as $tseq =$'$0.I$'.

3: Let the time between two consecutive events t be an observation of random variable with exponential distribution with mean value q_i.

4: Simulate the new state j:

- If $q_i = 0$, set $j = i$ and stop;
- If $q_i \neq 0$, simulate a discrete random variable with probability distribution given by the i-th row of the Π-matrix, i.e, $\frac{q_{ij}}{q_i}$ with $j \neq i$;
- Convert j to a letter, say J, and let $tseq = tseq+$ '$,t.J$'.

5: Return to Step 3.

We used this data generating process to design two different experiments, detailed in the next sections, to test AliClu. Two remarks are of notice. First, we skip the first step of AliClu, concerning temporal sequences generation from raw data, for obvious reasons. Second, as true cluster labels are known a priori, an additional analysis is made where clustering indices are computed between the true labels, i.e. the original parameter sets used to generate the data, and the clusters found by AliClu. In these experiments AliClu is run automatically.

4.2 Two clusters from the same two-state transition diagram

Two different clusters were generated from CTMCs with transition diagram given by Figure 10, cluster 1 with $\lambda_1 = 1000$ and cluster 2 with $\lambda_2 = 1$. Each cluster is composed by a number of sequences given by N. The goal is to separate correctly the clusters in the generated data.

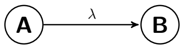

Figure 10: Transition diagram to generate temporal sequences with two clusters, each with a different λ.

For each number of sequences ($N = 5, 15, 25, 50$ and 100) and linkage function (single, complete, average, centroid, and Ward), experiments were repeated 25 times in order to obtain the percentage of correct decisions; this is defined as the number of times the AliClu method outputs the correct number of clusters divided by the total number of experiments. Results are presented in Figure 11.

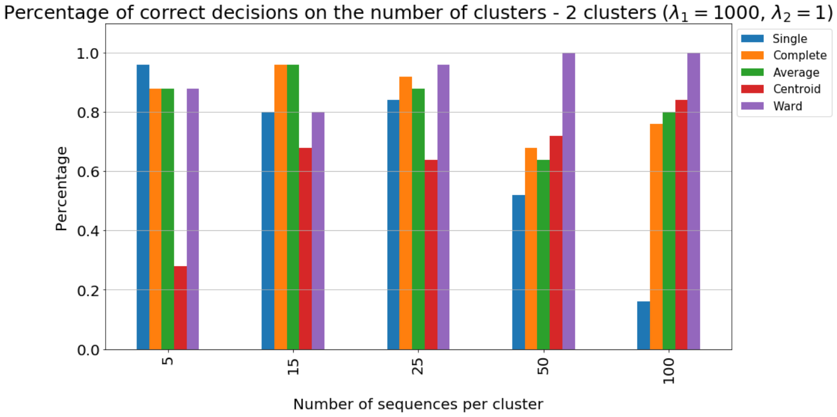

Figure 11: Percentage of correct decisions on the numbers of clusters by AliClu. Two clusters were sampled, from transition diagram in Figure 10, first cluster with $\lambda_1 = 1000$ and second cluster with $\lambda_2 = 1$.

It is possible to observe that linkage functions behave differently. Ward's method performed quite well, specially when the number of sequences is 50 and 100, where 100% of correct decision on the number of clusters were attained. Overall, the Ward's method has the best performance among all linkage functions. Table 5 presents statistics of the clustering indices – Rand, adjusted Rand (AR), Fowlkes and Mallows (FM), Jaccard and adjusted Wallace (AW) – between the original partitions and ones found with the Ward's method. Averages are quite high with low standard deviations. In addition, all measures agree, i.e. they present values close to each other and, therefore, it is possible to conclude that elicited clusters are equal to the original ones in the majority of the experiments.

4.3 Four clusters from different transition diagrams

Herein, data composed of four clusters generated from different CTMCs in Figure 12, were generated to assess AliClu. As before, the number of sequences N per cluster varies, taking values 5, 15, 25 and 50.

The percentages of correct decisions for different gap (g from -1.0 to 1.0 with incremental steps of 0.1) and temporal (T_p values of 0.25, 1, 1.5 and 2) penalties of the TNW algorithm are plot in Figures 13–16. Ward's method was used in the agglomerative algorithm of AliClu in all experiments. In Figure 13 it is possible to observe that the percentage of correct decisions is zero for the majority of gap penalties and N. However, by increasing the parameter T_p, the percentages of correct decisions increase, mainly for positive gap values, which is observed in Figure 14 for $T_p = 1$. Overall, a good performance is achieved

		Number of sequences per cluster				
		5	15	25	50	100
Average	Rand	0.982	0.984	0.967	0.978	0.980
	AR	0.963	0.967	0.933	0.956	0.959
	FM	0.980	0.983	0.966	0.978	0.980
	Jaccard	0.967	0.969	0.939	0.957	0.961
	AW	0.966	0.968	0.936	0.957	0.960
Standard Deviation	Rand	0.059	0.036	0.054	0.023	0.026
	AR	0.112	0.072	0.107	0.045	0.052
	FM	0.065	0.037	0.054	0.023	0.026
	Jaccard	0.106	0.067	0.093	0.043	0.046
	AW	0.110	0.069	0.100	0.044	0.049
Median	Rand	1	1	1	0.980	0.990
	AR	1	1	1	0.960	0.980
	FM	1	1	1	0.980	0.990
	Jaccard	1	1	1	0.960	0.980
	AW	1	1	1	0.960	0.980

Table 5: Average, standard deviation and median of five clustering indices when using Ward's method (c.f. Figure 11).

when $g = 0.3$ and $g = 0.4$; the latter presents the highest percentages for all N. In Figure 15, where $T_p = 1.5$, the method performed better from $g = 0.6$ to $g = 1$. The best result is achieved for $g = 0.8$ with 100% of correct decisions on the number of clusters for all N. A deterioration of the performance is observed again in Figure 16 where $T_p = 2$. As before, the percentages of correct decisions are mainly zero for the majority of gap values, as for the case with $T_p = 0.25$. Finally, we note that the clustering indices between the groups found and the original ones, for cases where AliClu performed best (Figures 14 with $g \in [0.3\ 0.5]$ and 15 with $g \in [0.7\ 0.9]$), validate that the correct clusters are obtained. Indeed, the medians and averages of the clustering indices are always one or almost one, whereas the standard deviations are zero or almost zero.

To conclude, it was shown that by increasing the parameter T_p, good performances can be obtained. The analysis of these synthetic data suggests that gap values used in AliClu should range over by positive gap values, rather than negative ones.

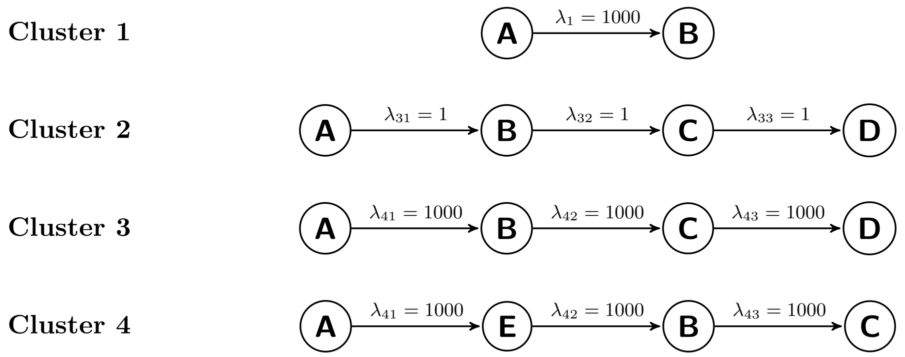

Figure 12: Transition diagrams to generate temporal sequences with four clusters.

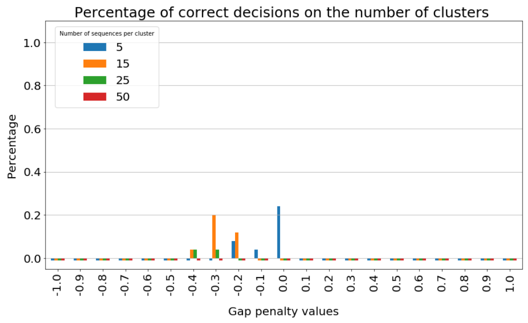

Figure 13: Results for $T_p = 0.25$.

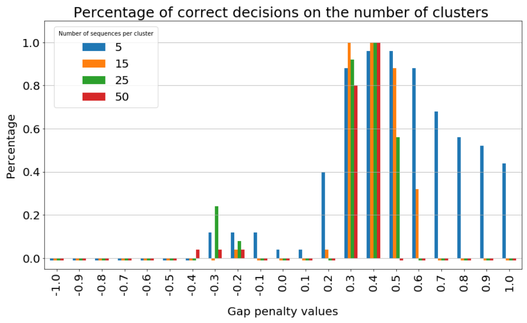

Figure 14: Results for $T_p = 1$.

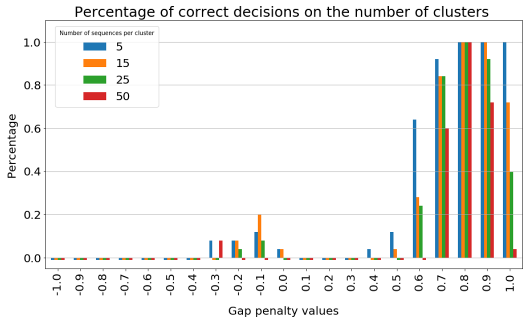

Figure 15: Results for $T_p = 1.5$.

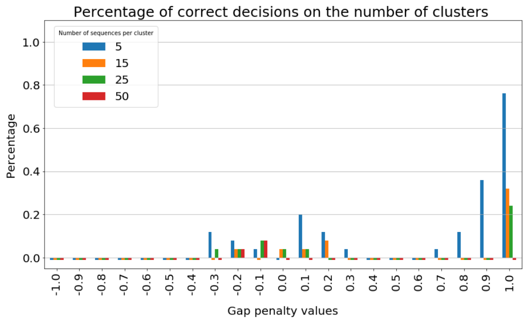

Figure 16: Results for $T_p = 2$.
